# Understanding perceptions of hair loss in hijab-wearing women: a pilot survey study

**DOI:** 10.1097/JW9.0000000000000115

**Published:** 2023-11-16

**Authors:** Lina Alhanshali, Fatima Bawany, Michael G. Buontempo, Jerry Shapiro, Kristen Lo Sicco

**Affiliations:** a Department of Dermatology, SUNY Downstate School of Medicine, Brooklyn, New York; b The Ronald O. Perelman Department of Dermatology, New York University Grossman School of Medicine, New York City, New York; c Department of Dermatology, Hackensack Meridian School of Medicine, Nutley, New Jersey

**Keywords:** hair loss, headwear, hijab, Muslim

**What is known about this subject in regard to women and their families?**

- The hijab is a head covering worn by millions of Muslim women throughout the world and has been associated with alopecia.

**What is new from this article as messages for women and their families?**

- Muslim women in this survey study self-reported alopecia related to the hijab.
- There are ways of mitigating the risks of alopecia or treating it without compromising religious beliefs or values.

## Dear Editors,

The hijab, a religious head covering worn by millions of Muslim women globally, has been the subject of various discussions, including its potential association with hair loss. Studies have explored the occurrence of traction alopecia associated with religious head coverings.^1–3^ Regarding hijab use and hair loss, 1 study reported that the top 27 YouTube videos on the topic have garnered over 17 million views.^4^ The scientific literature on hijab-associated alopecia remains sparse, with an absence of studies examining Muslim women’s perspectives on hair loss.

To bridge this gap, we conducted a 26-question electronic survey study, approved by the Institutional Review Board, among hijab-wearing Muslim women in New York City’s mosques, to comprehend their experiences with the hijab, alopecia, and healthcare-seeking behavior. The sample comprised 104 women, with a 90.6% response rate. The majority of the participants were between 18 and 25 years old (57.7%, *n* = 60/104), and nearly 90% had been wearing the hijab for 4 or more years (87.5%, *n* = 91/104) (Table 1).

**Table 1:** Styling practices and perceptions of hair loss in hijab-wearing women (*N* = 104)

Variable	*n* (%)
Hours per day of hijab wear	
<3 hours	3 (2.9)
4–6 hours	28 (26.9)
7–9 hours	34 (32.7)
10–12 hours	24 (23.1)
13–16 hours	10 (9.6)
17–20 hours	1 (0.1)
21–24 hours	4 (3.8)
Hairstyles worn under the hijab	
Low bun	76 (73.1)
Low ponytail	7 (6.7)
High bun	22 (21.2)
High ponytail	3 (2.9)
Braid	6 (5.8)
Other/no response	6 (5.8)
Use of undercap	
Yes	87 (83.7)
No	15 (14.4)
Other/no response	2 (1.9)
Felt that hair was very important to self-identity	74 (71.8)
Aware of hijab-associated hair loss	
Yes	65 (62.5)
No	39 (37.5)
Experienced hijab-associated hair loss	36 (34.6)
If experienced hair loss, duration of hair loss >12 months	22 (61.1)
Location of hair loss	*N* = 36
Frontal hairline only	14 (38.9)
Diffuse thinning only	6 (16.7)
Back of scalp	1 (2.8)
Sides of scalp only	1 (2.8)
Frontal + diffuse thinning	5 (13.9)
Frontal hairline + sides of scalp	4(11.1)
Frontal hairline+ back of scalp + diffuse thinning	1 (2.8)
Frontal hairline + sides of scalp + diffuse thinning	2 (5.6)
Strategies used to prevent or treat alopecia	
Topical therapies	22 (21.2)
Oral therapies	8 (7.7)
Changing hijab or hair styling practices	31 (29.8)
Comfortable seeking care from a medical provider	80 (76.9)
Dermatologist	59 (56.7)
Primary care provider	20 (19.2)
Other/no response	1 (0.1)
Barriers to seeking medical care for hair loss	
Lack of culturally competent care	15 (14.4)
Lack of provider knowledge regarding hijab-related practices	13 (12.5)
Lack of hijab-wearing medical providers	11 (10.5)

This table presents the results of a survey study conducted among 104 hijab-wearing Muslim women in New York City. It provides a detailed breakdown of the participants’ daily hijab-wearing habits, hairstyles, and their perceptions and experiences of hair loss associated with hijab wear. The variables are presented alongside the number and percentage of participants who reported each response. For the variable “Location of hair loss,” the total number of respondents was 36, as this question was only applicable to those who reported experiencing hijab-associated hair loss.

In terms of styling practices, over two-thirds of the participants wore the hijab for at least 7 hours daily (70.2%, *n* = 73/104). Approximately 85% (83.4%, *n* = 87/104) wore an undercap beneath their hijab. Interestingly, nearly two-thirds of the participants were aware of potential alopecia associated with the hijab (62.5%, *n* = 65/104). Over one-third reported experiencing hair loss that they attributed to the hijab (34.6%, *n* = 36/104) and of those, 26 (72.2%) identified the frontal hairline as the location of the hair loss, either exclusively or in combination with other scalp areas.

Significantly, 71.2% of women (*n* = 74/104) reported that hair is integral to their self-identity. Most women expressed willingness to seek medical care for hair loss (76.9%, *n* = 80/104). However, 29.8% (*n* = 30/104) identified barriers to seeking medical care, most frequently a perceived lack of cultural competency and knowledge of hijab-related practices among healthcare providers.

Our study reveals that a significant number of Muslim women experience alopecia they believe is associated with hijab wear. We found that hair is a crucial aspect of Muslim women’s identities, yet over one-third of the surveyed women were unaware of hijab-associated alopecia. This underscores the vital role of healthcare providers in educating women about religious headwear-associated traction alopecia and its mitigation to prevent potentially irreversible effects.

Healthcare providers should proactively offer strategies for traction alopecia mitigation to their hijab-wearing patients such as adopting looser hairstyles, altering hijab fabrics (jersey or cotton is less likely to slip), or the use of undercaps with ties to allow for size adjustment. If hair under hijab is styled in a knot or ponytail, varying the location in which it is styled is recommended. Medical therapies can be considered when alopecia is present. Such recommendations can help prevent and treat hair loss without compromising a patient’s religious practices or beliefs.

Study limitations include a small sample size, self-reported alopecia, and the absence of a comparison to a group of non-hijab-wearing women. Additionally, the data was exclusively collected from women in mosques, potentially biasing the results as these women may be more religious and discussions on hijab-associated alopecia may be a particularly sensitive topic. Finally, this study on a United States-based population may not be generalizable to the global population. Factors such as differences in hair type, pin placement, and hair-cutting practices may influence results. Despite these limitations, this data provides valuable insights into Muslim women’s perceptions of hair loss, enabling healthcare providers to address their needs more effectively. Controlled prospective studies to objectively evaluate this population are necessary.

## Conflicts of interest

J.S. is a consultant for Aclaris Therapeutics, Incyte, and Replicel Life Sciences. J.S. and K.L.S. have been investigators for Regen Lab and are investigators for Pfizer. K.L.S. is a consultant for Pfizer and Aquis. The other authors have no conflicts of interest to disclose.

## Funding

None.

## Study approval

This study was approved by the IRB at New York University Grossman School of Medicine (s23-00087).

## Author contributions

LA: Participated in research design, performance of the research, data analysis, and writing of the manuscript. FB: Participated in research design, performance of the research, data analysis, and writing of the manuscript. MGB: Participated in research design, performance of the research, and writing of the manuscript. JS: Participated in research design, performance of the research, data analysis, and writing of the manuscript. KLS: Participated in research design, performance of the research, data analysis, and writing of the manuscript.

## Data availability

Data is incorporated into the article. Additional data is available on request.
